# Reducing the smoking-related health burden in the USA through diversion to electronic cigarettes: a system dynamics simulation study

**DOI:** 10.1186/s12954-021-00484-6

**Published:** 2021-03-20

**Authors:** Arielle S. Selya

**Affiliations:** 1 Behavioral Sciences Group, Sanford Research, 2301 East 60th Street North, Sioux Falls, SD 57104 USA; 2 Department of Pediatrics, University of South Dakota Sanford School of Medicine, 1400 West 22nd St, Sioux Falls, SD 57105 USA; 3 System Dynamics Group, Department of Geography, University of Bergen, Postboks 7802, 5020 Bergen, Norway; 4 Pinney Associates, Inc, 201 North Craig St. Suite 320, Pittsburgh, PA 15213 USA

**Keywords:** Combustible cigarettes, Electronic cigarettes, Health policy, Nicotine, Smoking, Simulation modeling, System dynamics, Tobacco policy

## Abstract

**Background:**

Electronic cigarettes (“e-cigarettes”) have altered tobacco smoking trends, and their impacts are controversial. Given their lower risk relative to combustible tobacco, e-cigarettes have potential for harm reduction. This study presents a simulation-based analysis of an e-cigarette harm reduction policy set in the USA.

**Methods:**

A system dynamics simulation model was constructed, with separate aging chains representing people in different stages of use (both of combustible cigarettes and e-cigarettes). These structures interact with a policy module to close the gap between actual (simulated) and goal numbers of individuals who smoke, chosen to reduce the tobacco-attributable death rate (i.e., mostly combustible cigarette-attributable, but conservatively allowing e-cigarette-attributable deaths) to that due to all accidents in the general population. The policy is two-fold, removing existing e-liquid flavor bans and providing an informational campaign promoting e-cigarettes as a lower-risk alternative. Realistic practical implementation challenges are modeled in the policy sector, including time delays, political resistance, and budgetary limitations. Effects of e-cigarettes on tobacco smoking occur through three mechanisms: (1) diversion from ever initiating smoking; (2) reducing progression to established smoking; and (3) increasing smoking cessation. An important unintended effect of possible death from e-cigarettes was conservatively included.

**Results:**

The base-case model replicated the historical exponential decline in smoking and the exponential increase in e-cigarette use since 2010. Simulations suggest tobacco smoking could be reduced to the goal level approximately 40 years after implementation. Implementation obstacles (time delays, political resistance, and budgetary constraints) could delay and weaken the effect of the policy by up to 62% in the worst case, relative to the ideal-case scenario; however, these discrepancies substantially decreased over time in dampened oscillations as negative feedback loops stabilize the system after the one-time “shock” introduced by policy changes.

**Conclusions:**

The simulation suggests that the promotion of e-cigarettes as a harm-reduction policy is a viable strategy, given current evidence that e-cigarettes offset or divert from smoking. Given the strong effects of implementation challenges on policy effectiveness in the short term, accurately modeling such obstacles can usefully inform policy design. Ongoing research is needed, given continuing changes in e-cigarette use prevalence, new policies being enacted for e-cigarettes, and emerging evidence for substitution effects between combustible cigarettes and e-cigarettes.

## Introduction

Smoking tobacco is a causal factor in a wide range of adverse health effects including cancers [1–3], cardiovascular disease [4], macular degeneration [5], birth defects [6], rheumatoid arthritis [7], inflammation [8], and impaired immune function [9], and exacerbates diabetes [10, 11]. Through a combination of public policy (e.g., cigarette taxes, age restrictions on purchasing, and bans on advertising to youth) and increased public awareness of the health dangers of smoking, the USA has had success in drastically reducing the smoking prevalence over the past several decades, from 42% in 1965 to about 16% currently [12]; however, recent years show this reduction hitting a plateau [13], possibly due to “hardening” of remaining smokers as detailed below, implementation gaps in existing policies [14], or barriers to smoking cessation among vulnerable groups [15]. As a result, reductions in smoking-related morbidity and mortality have stagnated, and cigarette smoking remains the primary cause of preventable death and disease in the USA [12].

Adverse health outcomes attributable to smoking are primarily due to combustible elements of cigarettes [16, 17] as well as tars and carbon monoxide [18]. For example, lung cancer is perhaps the most well-known health risk of cigarette smoking, and approximately 90% of cases are attributable to smoking [12]. Despite reductions in smoking prevalence, the incidence of lung cancer has remained high (incidence rates of 100 per 100,000 in 1980, with only a slight reduction to 70 cases per 100,000 in 2010) [12]. Even more concerning, combustible cigarettes seem to be becoming more harmful over time, at least for lung cancer: despite the declines in smoking prevalence, lung cancer incidence as well as mortality has increased among those who smoke, particularly in adenocarcinoma. The increased cancer burden is speculated to be due to changes in the composition and processing of combustible cigarettes [12]. This increase in lung cancer rates runs counter to the decline in smoking prevalence over the last several decades.

Although nicotine is the major, but not only addictive component in combustible cigarettes [19], nicotine itself is not much more harmful than caffeine [18] as evidenced by low risk profiles of nicotine replacement therapy (NRT) products such as nicotine patches and gum [18]. Given the vastly different risk profile of nicotine alone versus other constituents in cigarette smoke, a great deal of harm reduction can be achieved by encouraging individuals who smoke to transition away from tobacco cigarettes to noncombustible nicotine products such as e-cigarettes [18]. Though some tobacco control advocates including a US Surgeon General have argued for heavily regulating all nicotine products [20], studies supporting the “hardening hypothesis” [13, 21, 22] raise doubts that nicotine use can be eliminated entirely. That is, the population of smokers in recent years have higher levels of nicotine dependence [13, 22] and higher rates of mental health comorbidities [21], relative to in the past, suggesting that today’s smokers are the remaining “hardened” group who face greater difficulties in stopping smoking. For example, over 60% of individuals suffering from schizophrenia smoke, which may be due to using nicotine to self-medicate their symptoms [23]. Taken together, diverting individuals who smoke combustible cigarettes to other sources of nicotine is a valid and likely effective harm reduction strategy.

Electronic cigarettes (e-cigarettes) are an alternate tobacco product that electronically heats e-liquid (a solution that can contain nicotine and/or flavorings) into vapor and thus lacks the harmful constituents in tobacco smoke [24]. E-cigarettes first appeared on the market around 2010 and have continually increased in popularity, resulting in more youth ever vaping nicotine (44.3% of 12^th^-graders) than ever smoking cigarettes (24.0%) according to Monitoring the Future 2020 (REF) [25]. Though long-term health data on e-cigarettes will not be available for some time, they are estimated to be only 5% as harmful as combustible cigarettes [24] and thus represent an important and appealing harm reduction alternative [18]. Many established smokers use e-cigarettes to offset or quit cigarette smoking [26–29], and despite not being approved for this purpose by the Federal Drug Administration (FDA) in the USA, e-cigarettes may be more effective than NRT for cessation [30].

A special consideration in smoking harm reduction is adolescents who are nicotine-naïve. Though recent research supports e-cigarette use as a harm reduction method among established, nicotine-dependent smokers who have difficulty quitting [31], much literature has encouraged restricting youth access to and interest in e-cigarettes, for example via e-liquid flavor bans [32, 33]. The motivation for restricting adolescent e-cigarette use stems from fears of e-cigarettes acting as a “gateway” to tobacco use, including combustible cigarettes [34, 35]. Evidence for the gateway mechanism includes adolescents who use e-cigarettes being at much higher risk for subsequent smoking relative to adolescents who have not used e-cigarettes [35, 36]; however, these adolescents have pre-existing risk factors that predisposed them to smoking, suggesting they would have gone on to combustible cigarettes anyway [37, 38]. Population trend modeling studies have also raised doubts about a gateway effect, as declines in smoking among youth have accelerated after the appearance of e-cigarettes [39, 40]. This suggests a possible “primary prevention” effect of e-cigarettes, which has been understudied [41] but is supported by recent studies, showing that e-cigarettes may be diverting adolescents from ever using combustible cigarettes [42, 43].

The current study investigates the potential of a harm reduction policy of promoting e-cigarette use in order to divert those who currently smoke or would otherwise smoke away from combustible cigarettes, using system dynamics simulation modeling. The model developed here is set in the USA, though it can easily be adapted to other settings by re-calibrating relevant parameters. System dynamics modeling is used to first replicate historical trends in youth use of combustible cigarettes and e-cigarettes, and then to project trends into the future under base-case scenario and policy scenarios. The policy acts through two mechanisms: removing existing flavor bans on e-cigarettes (as one method of increasing access to e-cigarettes), and a public health marketing campaign promoting e-cigarettes as a lower-risk alternative to combustible cigarettes (e.g., to correct growing misperceptions of relative risk of e-cigarettes [44, 45], given that perceived risk correlates with subsequent use [46]). Effects of this policy on the model are assumed to be threefold: (1) diverting nicotine-naïve adolescents from ever using combustible cigarettes; (2) reducing smoking behavior among existing smokers; and (3) increasing smoking cessation. The model was calibrated using data from the US National Youth Tobacco Survey (NYTS) [47, 48]. Long-term outcomes of cigarette smoking prevalence are examined as a function of different policy variants.

## Methods

### Causal loop diagram

**Fig. 1:**
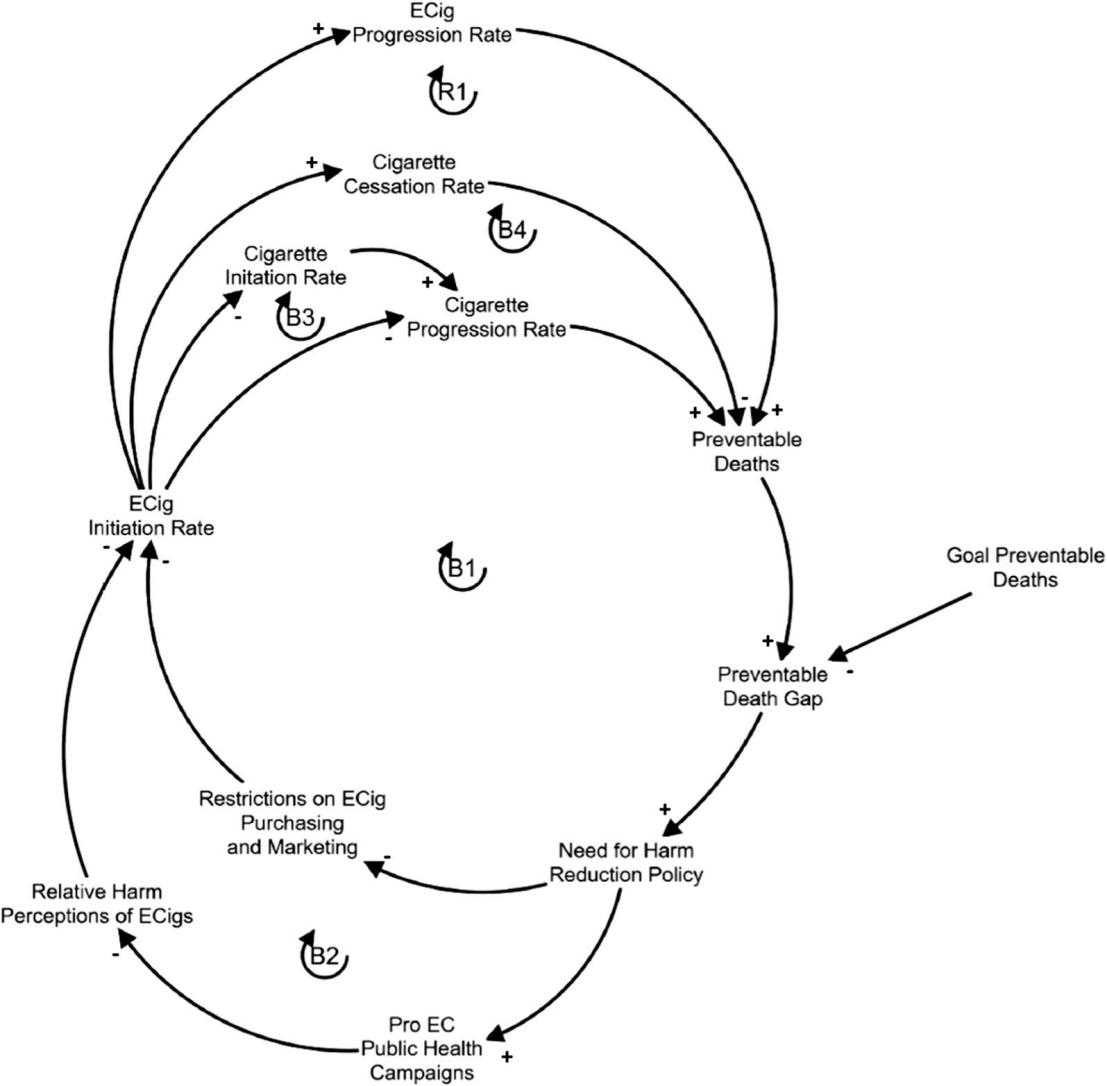
Causal loop diagram of the major feedback loops in cigarette smoking, e-cigarette use, and the current policy. Arrows denote causal links, and the polarity (± , next to each arrow head) denotes a positive relationship and an inverse relationship, respectively. Loops are denoted with an R (reinforcing/positive reinforcing loop) or B (balancing/negative reinforcing loop) and are numbered accordingly. The policy is twofold (public health campaigns that promote e-cigarettes as a less harmful alternative to cigarettes; removing restrictions on e-cigarette purchasing (here, removing e-liquid flavor bans)). E-cigarettes have 3 possible intended effects on cigarette smoking (diversion from initiation, offsetting consumption, and increasing cessation) and one possible unintended effect (deaths from e-cigarette use, since they are not completely safe)

A causal loop diagram showing the minimal essential set of relationships describing combustible cigarette use, e-cigarette use, their hypothesized relationship between them, and the policy under examination is shown in Fig. 1. This conceptual diagram was used to guide the development of the subsequent simulation model (see below). The causal loop diagram shows primarily feedback loops, either positive/reinforcing (whereby a change in one variable accelerates its own future change through the chain of causal relationships) or negative/balancing (whereby a change in one variable limits its own future change).

Loops B1 and B2 illustrate the central dynamic in this model: that established combustible cigarette use leads to an unacceptable number of preventable deaths (relative to some goal). The term “tobacco-related deaths” is used here as an umbrella term meant to include possible deaths from both types of tobacco products (combustible cigarettes and e-cigarettes); though e-cigarettes are not derived from tobacco, this terminology reflects the FDA’s designation of both e-cigarettes and combustible cigarettes as tobacco products. The unacceptably high number of tobacco-related deaths (which is nearly all due to combustible cigarettes) motivates a harm reduction policy, which in this case is twofold: (1) implementing an informational campaign which promotes e-cigarettes as a less risky alternative to combustible cigarettes (B1); and (2) removing existing e-liquid flavor bans (which are in effect in many places) (B2). It is assumed that this, in turn, increases e-cigarette initiation. Increasing e-cigarette initiation is predicted to have 3 intended effects: (1) decreasing the combustible cigarette progression rate by offsetting combustible cigarettes with e-cigarettes (B1), (2) reducing the combustible cigarette initiation rate by diversion away from combustible cigarettes (B3), and increasing the smoking cessation rate (B4). An important potential unintended consequence is also included, which reflects the pessimistic possibility that established e-cigarette could theoretically increase the preventable deaths (though not as much as combustible cigarettes), which can counteract the policy to some degree (R1). Importantly, though there are no established cases of nicotine-vaping-related deaths to date, building this possibility into the model allows simulations under conservative, worst-case (within realistic estimates) assumptions, thus making simulation results more robust. That is, if e-cigarettes are found to be less than 5% as harmful as combustible cigarettes, the current results would conservatively *underestimate* the benefits of the current study’s harm reduction policy.

### Stock-and-flow diagram

**Fig. 2:**
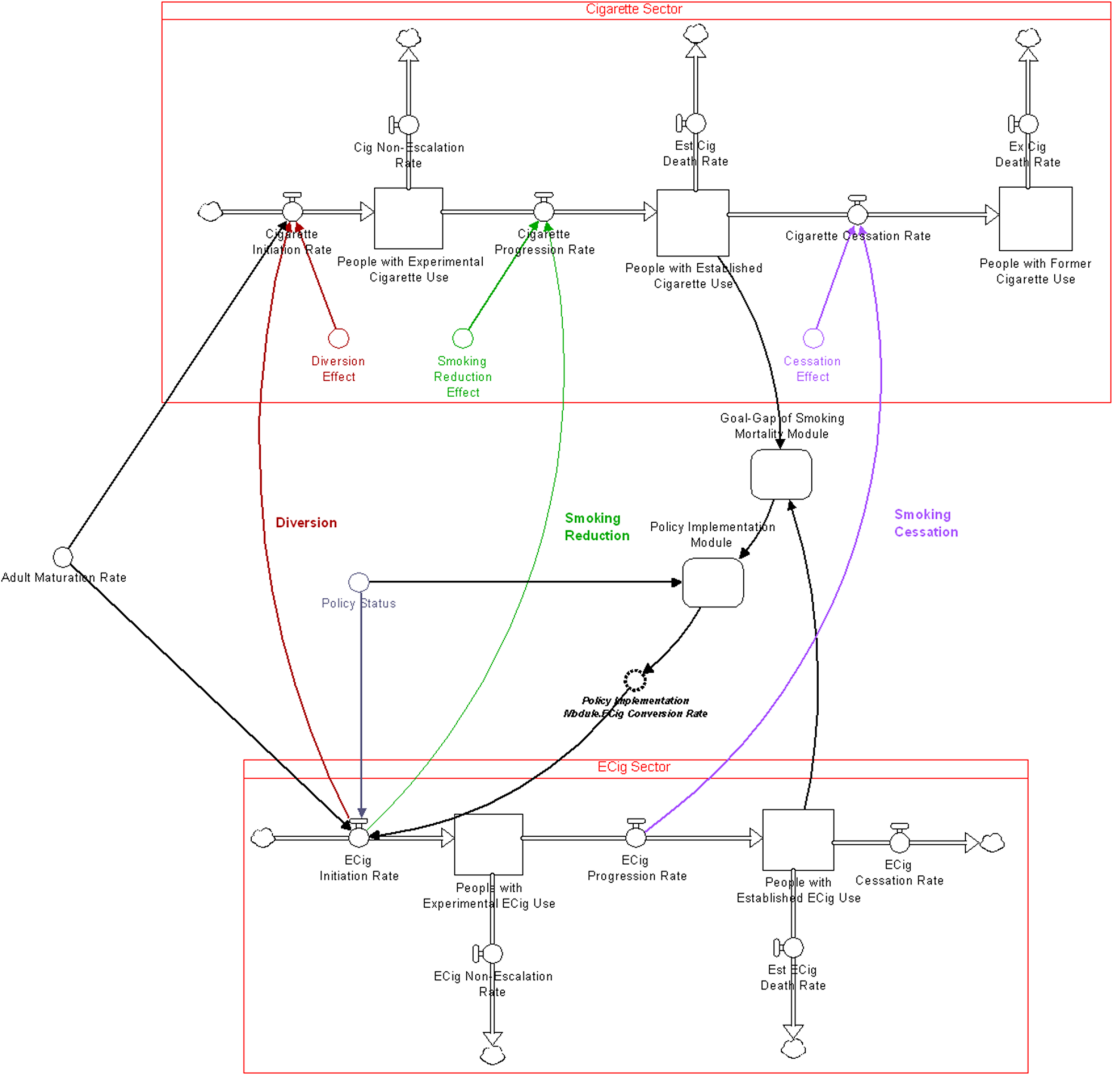
Simplified structure of the stock-and-flow model. Separate aging chains represent cigarette use (top sector) and e-cigarette use (bottom sector) with stocks (represented as squares) for different stages of use, and corresponding flows (represented as valves). The stock of individuals with “established cigarette use” and “established e-cigarette use” drives mortality, and this motivates policy implementation which targets e-cigarette initiation rate. This policy acts in 3 ways: diversion from cigarette initiation, reduction in the smoking progression rate, and increasing the cigarette cessation rate

Based on the CLD above, a stock-and-flow diagram (Fig. 2) was constructed in Stella Architect, version 1.9.5 [49], which consists of “aging chains” for both combustible cigarette and e-cigarette use (a structure consisting of stocks in series, here representing different stages of use, with appropriate inflows and outflows, representing transition rates). Each aging chain has an initial inflow dependent on the rate of individuals maturing into adolescence (i.e., turning 12 years old), which occurs continuously throughout the length of the simulation. The combustible cigarette aging chain has three stocks: people who are “experimenting” with cigarette smoking, people with “established” cigarette use, and people who “formerly” smoke cigarettes”; while the e-cigarette aging chain only has the first two. Individuals who previously used e-cigarettes were intentionally excluded from the model because there is lack of available data on this group to calibrate parameters. Instead, a simplifying assumption was made (e.g., due to the hardening hypothesis) that once a person establishes e-cigarette use, that they will always use e-cigarettes (i.e., we assume conservatively that they never quit using e-cigarettes). This is a conservative assumption (see Limitations). The flows from one stock to another are assumed to encompass two mechanisms: 1) a “social-recruitment” mechanism, in which the new initiation rate is positively affected by the proportion of individuals who currently smoke and/or use e-cigarettes; and 2) a “self-recruitment” mechanism in which there is a stable base of individuals who use nicotine products regardless of usage in the population, consistent with the hardening hypothesis [13]. The social recruitment mechanism parameter was set to 2, meaning that each individual who uses nicotine products influences 2 other individuals per year (see Calibration Testing below).

The two aging chains feed into the Goal-Gap of Tobacco-Related Mortality Module, which calculates the discrepancy between the actual tobacco-related deaths (from both combustible cigarettes and e-cigarettes, based on the respective stocks of individuals with established use, as this is the relevant measure from a health perspective [50]) and a goal value. Since it is unrealistic to entirely eliminate tobacco-related deaths, a goal value approximating the death rate from accidents of any type in the general population was chosen (1%/year), below the death rate due to poor diet/physical inactivity and alcohol consumption [51]. The discrepancy in the actual versus desired deaths in turn affects the policy implementation, as a much higher-than-desired tobacco-related death rate motivates regulatory policy.

The policy itself, captured in the Policy Implementation Module, consists of removing existing e-liquid flavor bans (as one type of policy which increases access to e-cigarettes) as well as an educational campaign promoting e-cigarettes as a less harmful alternative. The effect of the policy is modeled as increasing the e-cigarette initiation rate, which has a 3-pronged effect on the combustible cigarette aging chain: increasing diversion away from combustible cigarette initiation, reducing smoking and thus progression rates to established use, and increasing smoking cessation. E-cigarette initiation rate can be increased beyond that of combustible cigarette initiation, indicating that the model allows for nicotine-naïve individuals to initiate e-cigarettes (i.e., “gateway” effects); the e-cigarette and combustible cigarette initiation rates are both capped at the maximum of individuals becoming adolescents each year (maturation rate into adolescence). The Policy Implementation Module includes pragmatic considerations, such as political resistance to overturning an e-liquid flavor ban, and resources for implementing an informational campaign (both budgetary and workforce-related). The structure of this model allows for ideal, best-case scenarios (no resistance and sufficient resources) as well as more realistic, limited scenarios through changing corresponding parameters (i.e., the likelihood of removing an e-liquid flavor ban; the proportion of required funding that is approved, and time delays). The overall initiation of the policy is linked to a binary “switch” that can be turned on or off. It is important to note that, while the decision to initiate the policy is a binary variable, the policy itself once switched “on” can still cover a range of specific implementations, including uncertainty of further approval in the case of flavor bans on e-liquids.

The model was run for the period 2000–2100, with e-cigarettes first appearing around 2010, and the policy also being implemented in 2010. The detailed model structure and equations, including the modules, can be downloaded for free [52]; the model can be opened and run using the free software isee Player [53].

### Model calibration

The model was calibrated to match the “behavior modes” (i.e., the fundamental shape of the trend, such as exponential growth or exponential decline) observed in youth combustible cigarette and e-cigarette use in the USA over the period 2000–2019 (most recently available data), based approximately on the National Youth Tobacco Survey (NYTS) [47, 48] for dynamics relevant to youth (i.e., initiation and progression to established use) and the National Health Interview Survey (NHIS) [54] for dynamics relevant to adults (i.e., smoking-related fractional death rates). Since this model is not intended to finely replicate historical behavior or provide precise future projections, calibration to a broad behavior mode was sufficient. Some parameters were selected based on external data (lifetime probability of quitting combustible cigarettes, self-recruitment effect; experimenting and cessation rates; combustible cigarette-related *raw* death rates and life expectancies; cessation rates; diversion, smoking reduction, and cessation effects, times to established use; and all population numbers), while others (social contagion effects; combustible cigarette-related *fractional* death rates) were calibrated by running “live” simulations over a range of parameters to determine the optimal value with respect to stocks of individuals who smoke and who use e-cigarettes (“established users”), as these are the stocks relevant for public health. Stocks of individuals who smoke and individuals who use e-cigarettes according to NYTS [47, 48] and the NHIS [54] show approximately goal-seeking behavior towards a plateau (based on the proportion who are self-recruiters), and exponential growth for established e-cigarette use. Remaining parameters of flows (i.e., social contagion effect) were calibrated to achieve a reasonable match between simulated and historical data, based on the observed behavior mode and approximate magnitude (e.g., estimates of 46.5 million smokers in the USA in 2000 [55]. Specifically, the social contagion parameter was tuned over a wide range of plausible range values (0, meaning no social influence, to 10, meaning that each individual influences 10 other individuals), and the value that produces an initial decline followed by a plateau through the end of the time horizon (i.e., year 2100) was selected, consistent with the hardening hypothesis. Values higher than 2 (the final parameter value) produce a continuous decline, while values higher than 2 produce an *increase* in smoking prevalence, both of which are unrealistic.

Parameters for the three-fold hypothesized effects of e-cigarette use on combustible cigarette smoking (diversion, smoking reduction, and cessation) were quantified as follows. The diversion effect was quantified based on a separate study [42] as 55.4% of individuals who use e-cigarettes per year being diverted from initiating combustible cigarettes. No empirical estimates exist for a smoking reduction effect, and this was quantified using the rationale that, among adolescents who use either e-cigarettes or combustible cigarettes, about 30% preferentially use e-cigarettes historically [12, 18]; therefore, e-cigarettes may offset 30% of ever-smokers who progress to established smoking. The cessation effect was quantified based e-cigarettes having an approximately 20% success rate for smoking cessation in a randomized trial [56].

### Model validation

A range of validation tests were performed on the model, which identified errors that were corrected in the final model. Boundary conditions were examined conceptually to determine which variables and causal relationships were included in the model. Parameter assessment was based on external data sources and calibration to observed data. Extreme conditions testing was conducted by setting inflows and initial values of stocks to 0 and very high values, and ensuring the model behaved reasonably (e.g., stocks do not fall negative).

### Model analysis

A base-case model was constructed to replicate approximate trends in combustible cigarette and e-cigarette use among US adolescents and adults across 2000–2019 [40, 53, 54, 57] and projected into the future (year 2100). Several policy scenarios were run, including an ideal-world, best-case scenario (no practical obstacles to implementation), and scenarios where policy implementation is delayed, faces resistance (i.e., low likelihood of removing e-liquid flavor bans, due to controversy) and faces limited funding for an informational campaign. Specifically, time delays for the best-case scenario were set to 0.1 years (for time to approve both policies, time to approve funding, and time to hire and train workforce) and 10 years (for workforce turnover rate); and in the time-delayed model, were set to larger values (time to approve removal of e-liquid flavor bans = 2 years, time to approve informational campaign = 1 year, time to adjust workforce = 1 year, time to train workforce = 0.25 years, and time to approve funding = 1 year). With respect to uncertainty in approving the removal of e-liquid flavor bans, probability of approval was set to 1 and 0.5 for the ideal-case and uncertain-approval scenarios, respectively. With respect to budgetary constraints, the fraction of required budget that is approved is set to 1 (full budget) and 0.7 in the ideal-case and budget-restricted scenarios, respectively.

## Results

**Fig. 3:**
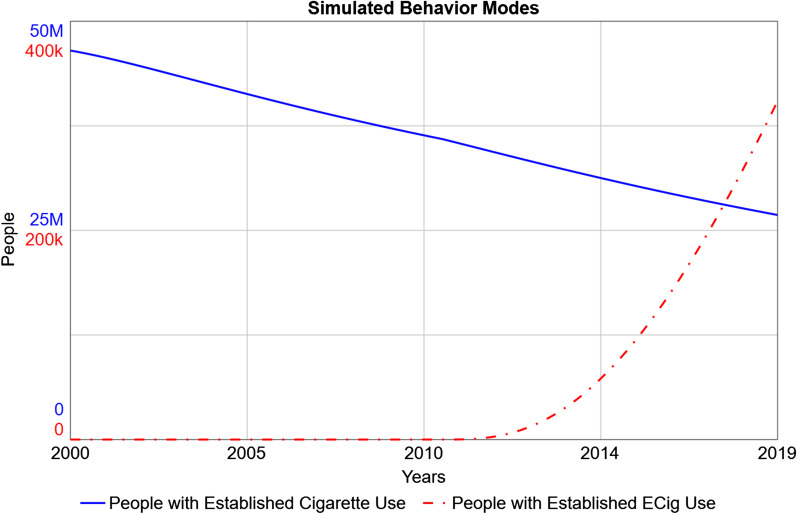
Simulated Behavior Modes for Exclusive Cigarette and E-Cigarette Use, 2000–2019. After model calibration, simulations predict approximately exponential decline in established cigarette use (solid blue line), and exponential increase in established e-cigarette use (dot-dashed red line), between 2000 and 2019 [57]. No policy is implemented in this simulation. Note the different y-axis scales for each line

The base model was able to successfully replicate the approximate behavior modes (Fig. 3) observed in historical data [29, 33] and NHIS [54], namely the slow, approximately exponential decline in established combustible cigarette use over 2000–2019 [57] and NHIS [54], and approximately exponential increase in established e-cigarette use from 2010 to 2019 [57].

**Fig. 4:**
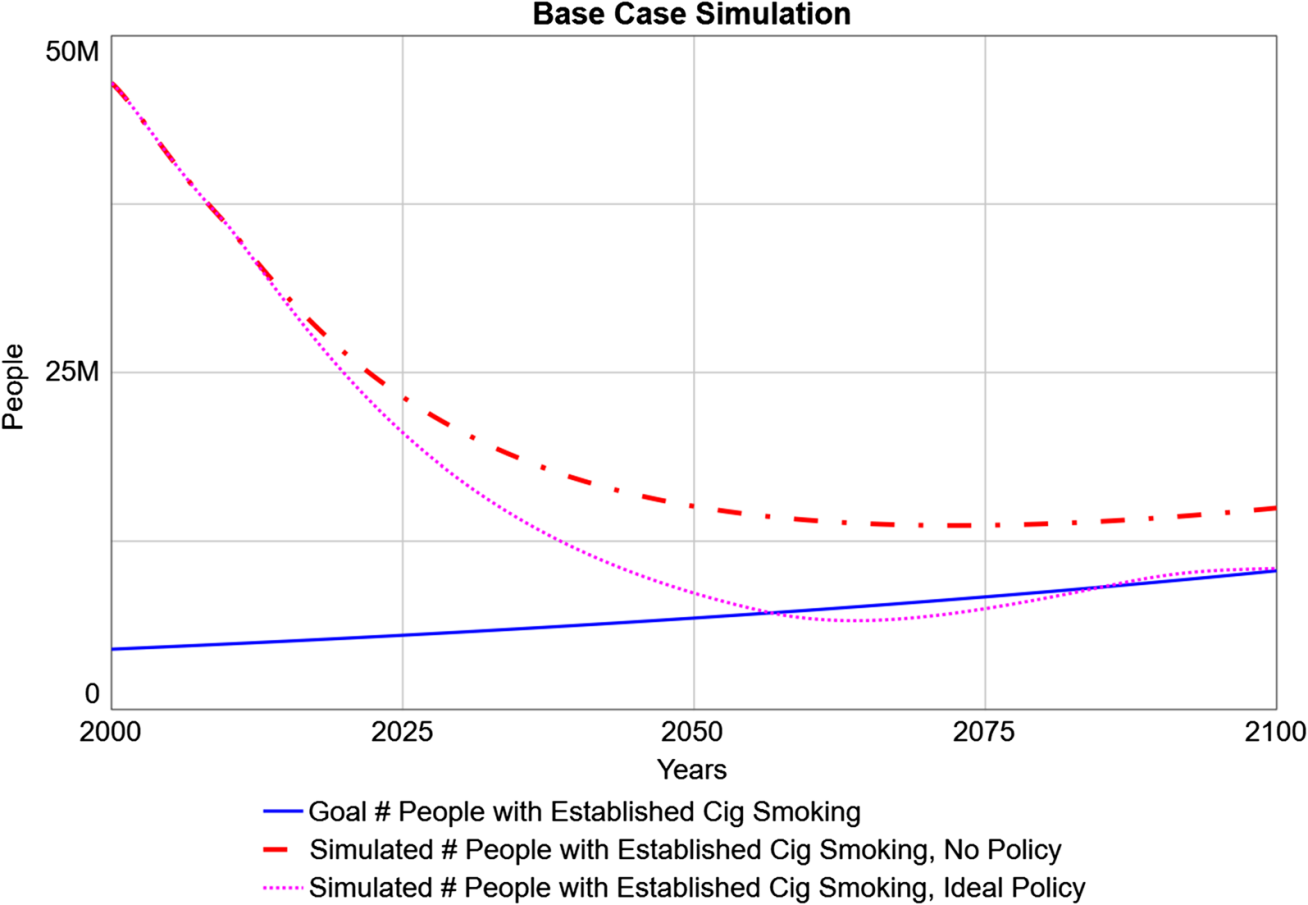
Simulated versus Goal Established Numbers of Individuals who Smoke. The goal number of people with established cigarette smoking (solid blue line) is based on the rate of accidental death in the general population, and increases over time with population growth. The simulated number of people with established cigarette smoking is shown in the status quo scenario (no policy; dot-dashed red line) and the ideal-policy scenario (starting in 2010; dotted pink line) throughout the time horizon under examination (through 2100)

Figure 4 presents the base-case simulation run through the year 2100, under the scenario of the status quo (no policy) and the ideal-case policy scenario relative to the goal number of established combustible cigarette smokers (which increases over time with population growth). Under the status quo, the simulated number of individuals who smoke declines, continuing the preexisting trend of exponential decline from 2000 to 2019; however, it remains higher than the target number. This persistent discrepancy leads to an unacceptably high number of preventable tobacco-related deaths throughout this time horizon. The ideal-policy scenario projects the policy (implemented in 2010) accomplishing its goal shortly after 2050, and subsequently exhibiting some minor oscillations around that goal. These oscillations are caused by delays in the implementation (e.g., workforce adjustment for the informational campaign); delays within negative feedback loops are well-understood in system dynamics to produce oscillations, as by the time decisions are fully implemented (e.g., hiring and training of staff), the adjustment needed to meet the goal has already changed due to pre-existing dynamics [58].

**Fig. 5:**
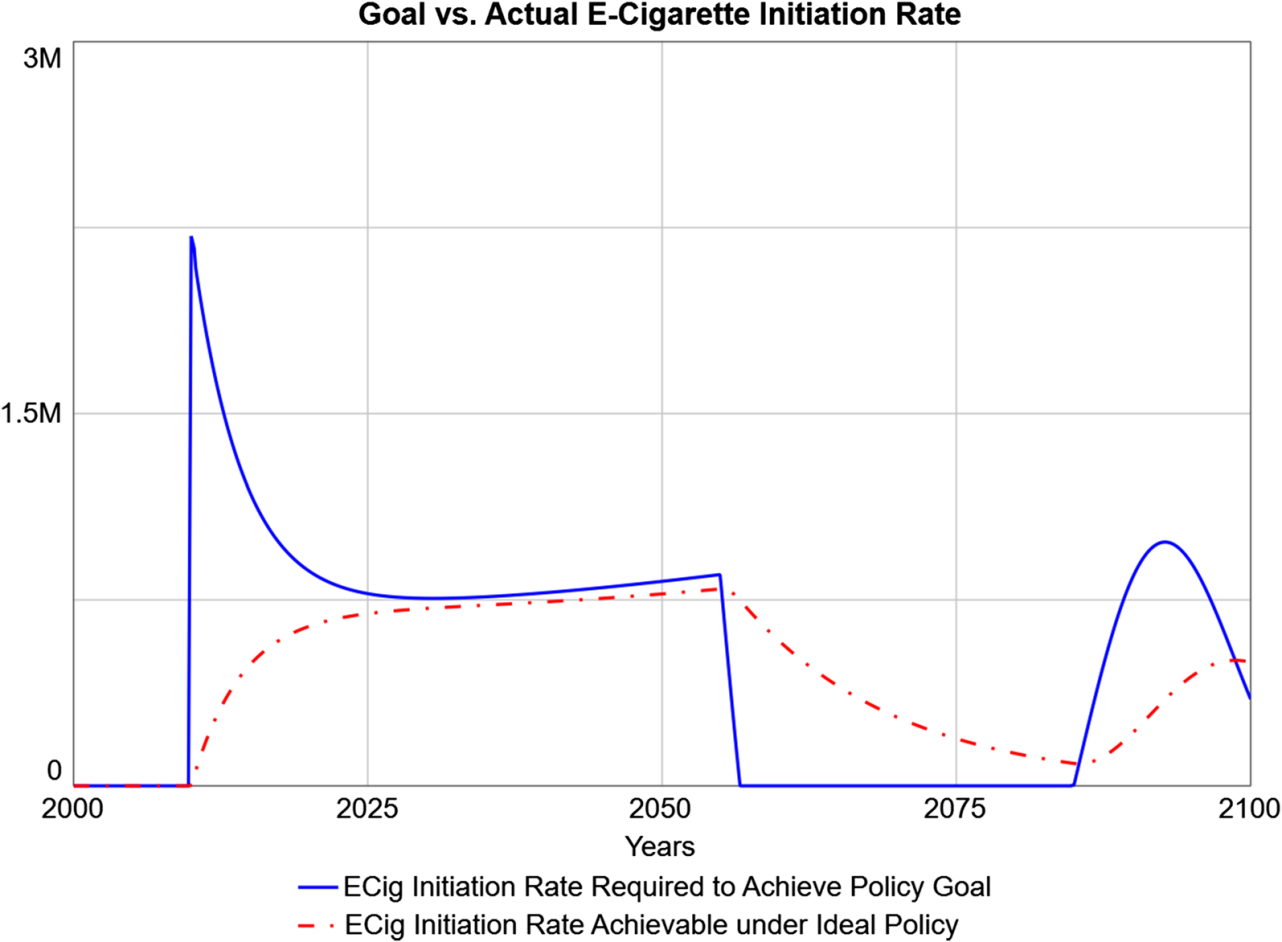
Simulated Optimal versus Achievable E-Cigarette Initiation Rates. The flow of e-cigarette initiation rates is the direct target of the policy. The e-cigarette initiation rate required to achieve the goal instantly (solid blue line) is shown against the e-cigarette initiation rate achievable within the limitations of the system (dot-dashed red line)

Figure 5 shows simulated e-cigarette initiation rates, since this is the flow being regulated by the policy. The desired initiation rate (i.e., the e-cigarette initiation rate required to achieve the policy goal) is presented against what is achievable within the practical constraints of the policy (e.g., time needed for individuals who may potentially use e-cigarettes to adjust their expectations) even if the policy was implemented with minimal delay, no political resistance, and full budgetary resources. The desired initiation rate is zero before 2010, as the policy is not yet implemented before then. The initial projected spike in desired e-cigarette initiation is due to the larger discrepancy between the actual and desired numbers of individuals who smoke in 2010, and the sudden enactment of the policy. This discrepancy is then projected to close as the actual number of individuals who smoke approaches its goal. However, the achievable e-cigarette initiation rate is projected to be slower and lacks the initial overshoot, as it takes time for individuals who may potentially use e-cigarettes to adjust their expectations about e-cigarettes and convert to use. Around 2060, the desired e-cigarette initiation rate suddenly is projected to drop to 0 because the stock of individuals who smoke crosses below the goal (see Fig. 4); desired e-cigarette initiation rate is projected to drop to 0 shortly after (with a lag due to the delays in the implementation module). In other words, the harm reduction policy is no longer needed, as the goal number of individuals who smoke has been met and surpassed; once this change is “registered” in policy, the desired e-cigarette initiation rate is 0, as further offsetting of combustible cigarette use is unnecessary.

**Fig. 6:**
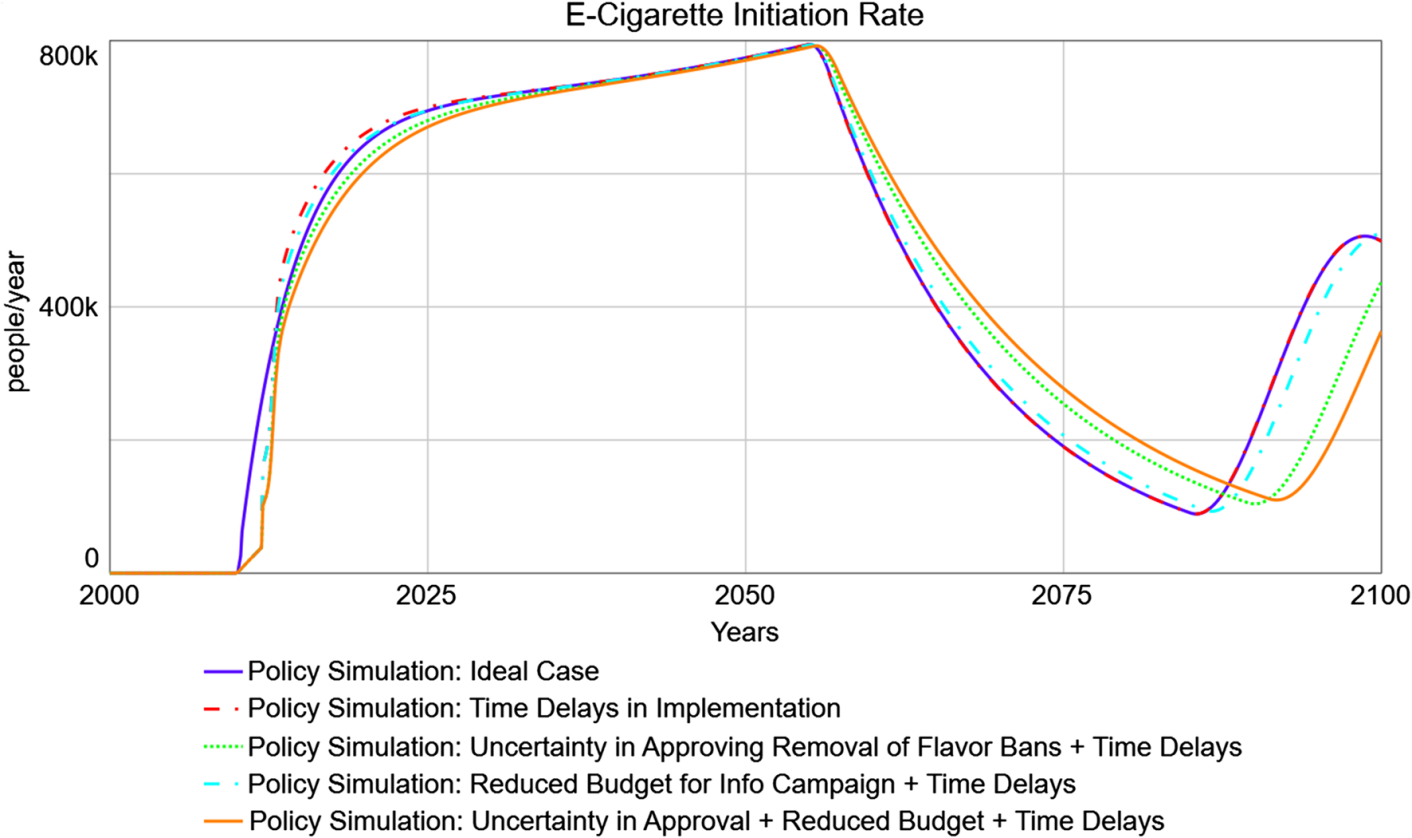
Policy Simulations of Predicted E-Cigarette Initiation Rates under Ideal Case versus Practical Limitations. The ideal case (solid blue line) is contrasted with policies with time delays in implementation, alone (dot-dashed red line), with uncertain approval for removing e-liquid flavor bans (dotted green line), with reduced budget for informational campaigns promoting e-cigarettes as a less harmful alternative (dot-dashed cyan line), and with both uncertain approval and reduced budget (solid orange line)

Figure 6 presents the simulated ideal-case e-cigarette initiation rate (i.e., the achievable rate from Fig. 5) against more realistic scenarios that include time delays to policy implementation and/or uncertainty in removing e-liquid flavor bans and reduced budgetary resources for an informational campaign. Compared to the ideal-case scenario, time delays in policy implementation are projected to produce a lagged predicted response in e-cigarette initiation rates accomplished by the policy, which is most severe at first, due to delay in policy approval. E-cigarette initiation rates then are predicted to overshoot the ideal-case scenario while maintaining a discrepancy with the ideal-case scenario, due to continuing delays in workforce adjustment related to the informational campaign. That is, due to the simulated delays in hiring and training workforce, current workforce lags desired workforce; and when desired workforce decreases, the actual workforce remains higher than necessary until the system adjusts again, temporarily resulting in higher-than-needed e-cigarette initiation rates. This lagged response of actual workforce behind desired workforce persists as long as the system is in disequilibrium [58]. A scenario with time delays in addition to budgetary constraints predicts similar behavior, with less overshoot in e-cigarette initiation rates relative to the ideal case during the first peak (through about 2055). In the subsequent undershoot, this scenario similarly predicts a dampened and delayed response compared to the ideal-case scenario, though this time in the opposite direction. A scenario with time delays in addition to uncertain approval of removing e-liquid flavor bans predicts a delay and consistent undershoot in e-cigarette initiation rates relative to the ideal-case scenario during the first peak, followed by similarly dampened and delayed responses during the subsequent undershoot. Finally, a scenario with all constraints (time delays, uncertain approval, and budgetary constraints) predicts a delayed and consistently undershooting e-cigarette initiation rate relative to the ideal case during the first peak, and a similar overshot and delay during the subsequent undershoot.

In order to quantify these differences between the ideal-case and scenarios that include realistic practical limitations, values from 2012 are examined (2 years after policy implementation, and the year of approximately greatest spread in e-cigarette initiation rates across scenarios). In the ideal case, approximately 265,000 people/year are projected to initiate e-cigarette use; versus approximately 161,000 and 160,000 in the time-delayed and time-delayed with reduced budget scenarios, respectively (a 39% difference relative to the ideal-case scenario at the same time point); and approximately 101,000 in both the time-delayed plus uncertain approval scenario, and the scenario with all three types of limitations (a 62% difference). This discrepancy is projected to close as time passes: all policies start to converge in 2050. However, oscillations are projected to persist into the future whenever the effective policy status changes (i.e., when established number of individuals who smoke oscillates past the goal value, and the need to actively encourage e-cigarette initiation is present or absent). These projected oscillations are dampened: for example, in the second phase of the first oscillation (approximately 2070), the ideal-case scenario projects that approximately 268,000 people/year will initiate e-cigarettes, versus 267,000 in the time-delayed scenario (a negligible difference relative to the ideal-case scenario at the same time point), 336,000 in the time-delayed plus uncertain approval scenario (a 25% difference), 286,000 in the time-delayed plus reduced budget scenario (a 7% difference), and 360,000 in the scenario with all three types of limitations (a 34% difference). Thus, the more time passes, the closer all scenarios are expected to become to each other, indicating that the system has recovered from the initial implementation obstacles.

**Fig. 7:**
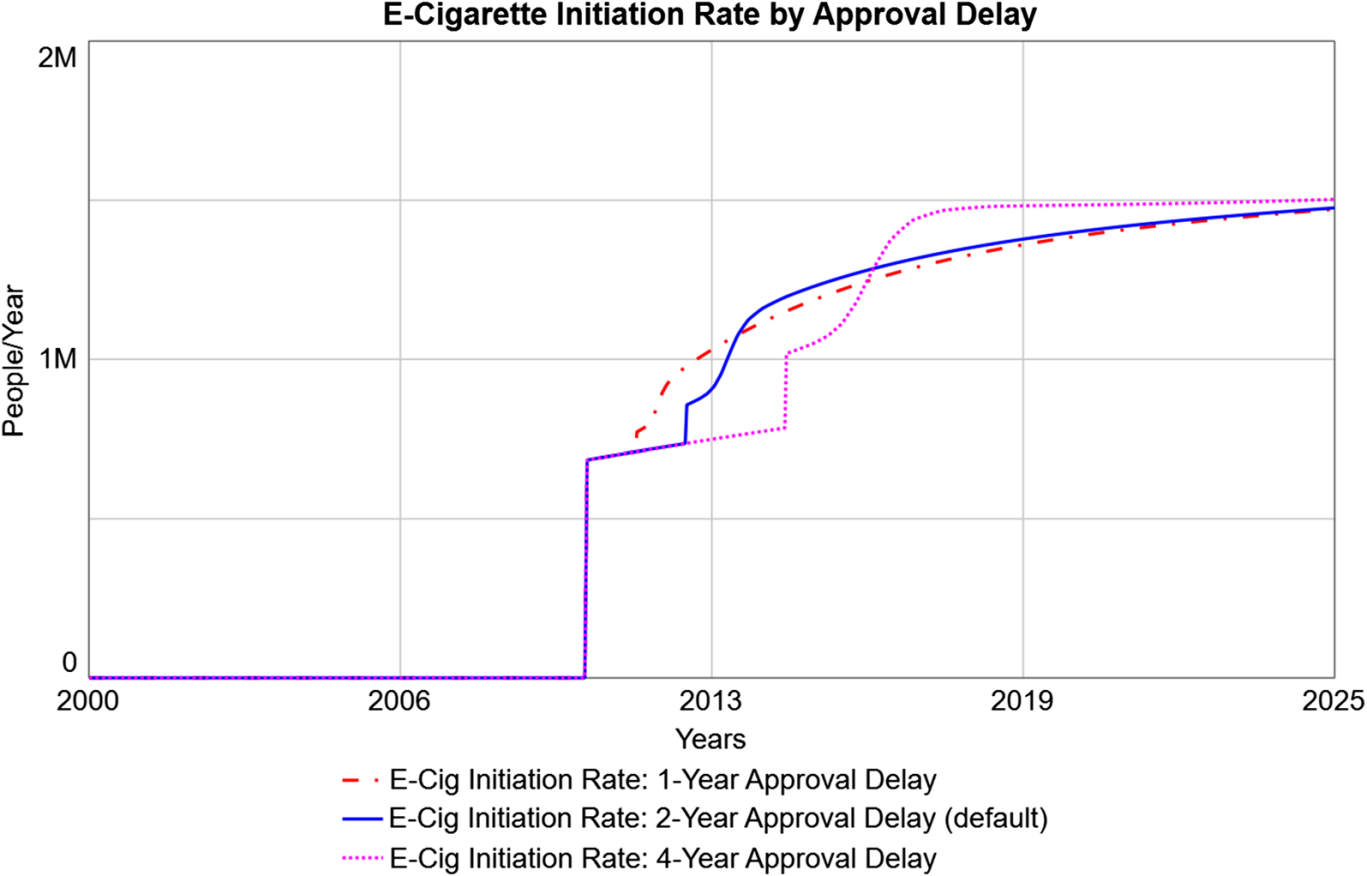
Predicted E-Cigarette Initiation Rates under Different Lengths of Approval Delay. The default delay in the model is 2 years for each policy (referring to a 2-year delay in revoking existing e-liquid flavor bans; and a 1-year delay in approving an informational campaign followed by a 1-year delay in approving the budget for the campaign), which proceed in parallel. The default 2-year delay (solid blue line) is shown in comparison with scenarios in which each of these delays is halved (dashed red line) or doubled (dotted pink line)

Finally, since the time delay required for legislative approval is uncertain, several simulations were run (Fig. 7) using the default delays (2 years for each policy, which proceed in parallel: this includes 2 years to approve overturning existing e-liquid flavor bans; and 1 year to approve the informational campaign followed by another year to approve the budget) as well as delays which take half as long (1 year) or twice as long (4 years). As the approval delays get longer, the time for projected e-cigarette initiation to increase is correspondingly delayed, and the increase has a steeper curve. This is due to the fact that more potential e-cigarette initiators are expected to be present by the time the policy takes effect.

With respect to the distal goal of reducing tobacco-attributed preventable deaths to the rate of accidental deaths in the general population, this policy is projected to achieve its goal around 2036 (26 years after policy implementation) (data available on downloadable model [52]), and subsequently the predicted preventable deaths remains far below the goal (2.7 million simulated deaths/year versus a goal of 4.5 million deaths/year in 2050). Notably, this figure includes the potential unintended consequence of preventable deaths attributable to e-cigarettes.

A user-friendly and interactive interface is publicly available on the web [59]. This interface version allows the individual to modify the above parameters related to implementation obstacles along their full possible ranges, as well as other parameters (namely, the hypothesized strength of the diversion, smoking reduction, and smoking cessation effects of e-cigarettes, across the possible range of each effect, i.e., 0 to 100% effect on the corresponding rate). In addition, the full model is also available for download [52] and can be viewed and run using the free software isee Player [53]. This can allow continued utility of this model as forthcoming data provide more precise estimates of relevant parameter values in the model.

## Discussion

This study presents a novel system dynamics model examining the promotion of e-cigarettes as a harm reduction policy towards the goal of reducing tobacco-related death and disease, which is primarily due to combustible cigarettes. This simulated policy, which acts through removing existing e-liquid flavor bans and implementing an informational campaign promoting e-cigarettes as a less harmful alternative, has a 3-pronged effect: 1) diverting adolescents from ever using combustible cigarettes; 2) reducing smoking among recent initiators, thereby lowering the progression rate to established combustible cigarette use; and 3) increasing smoking cessation. Policy simulations that promote e-cigarettes achieve a successful reduction in combustible cigarette smokers to the goal number, given the assumptions in this model. Achieving this goal would move combustible tobacco use from its current place as the leading behavioral cause of death, to below that attributable to poor diet/physical inactivity and alcohol consumption [51]. Realistic obstacles to policy implementation such as delays in decision-making, uncertain approval, and budgetary limitations have the effect of delaying and weakening the policy’s effects (by up to 62% in the first years after policy implementation, though these effects diminish over time). This system dynamics model is publicly available both as a full model [52] and useable via a user-friendly web format [59], allowing decision makers to test out the effects of different parameters and assumptions within a simulation setting.

E-cigarette policy remains a controversial topic. Some argue for strict regulation comparable to that of combustible cigarettes, based on health concerns and the addictive potential of nicotine. Strict regulation has often been justified for the sake of nicotine-naïve adolescents [20], due to fears of a “gateway” effect causing nicotine dependence and later combustible cigarette smoking [35] among youth who otherwise would not have smoked. However, given recent research supporting a common-liability hypothesis which postulates that the apparent relationship between e-cigarette use and smoking is attributable to a pre-existing liability for nicotine use [37, 38], the question of primary prevention becomes relevant [41]. That is, for youth who have a propensity to use nicotine, it is important to direct them to a less harmful product. Furthermore, tightening restrictions on e-cigarettes too strictly may have the unintended consequence of directing individuals who use nicotine products back to combustible cigarettes [60–62], which are more harmful due to the nature of combustible smoking [16, 18].

There is greater consensus that adults with extensive smoking history and severe nicotine dependence would be better off switching to e-cigarettes [18] due to their more favorable risk profile [24]. However, current and growing misperceptions about e-cigarettes’ risk relative to combustible cigarettes presents a barrier to switching to e-cigarettes among adults who smoke. Specifically, a majority of adults in the USA falsely believe that e-cigarettes are as harmful as, or more harmful than, combustible cigarettes, and this misperception is growing over time [44, 45]. In response to the recent E-Cigarette or Vaping-Related Lung Injury (EVALI) outbreak in the USA, harm perceptions of e-cigarettes increased when EVALI was initially and falsely attributed to e-cigarettes [63]; however, harm perceptions remained elevated even after EVALI was traced to vitamin E acetate, an agent in illicit tetrahydrocannabinol (THC) vape liquid [63]. Since risk perception of tobacco products correlates (negatively) with subsequent use, these misperceptions of e-cigarettes’ risk may be preventing adults who smoke from switching away from combustible cigarettes. This suggests that an informational policy as tested in this study may be effective for harm reduction; a similar approach has been taken by Public Health England [24].

Through simulation modeling, the current study suggests that a harm reduction policy promoting e-cigarettes could reduce smoking prevalence through a combination of diversion, smoking reduction, and cessation. In turn, the preventable, tobacco-attributed deaths eventually (after dampened oscillations) approach goal value (i.e., the rate of accidental deaths in the general population). This reduction in tobacco-attributable deaths remains substantial even after accounting for the important unintended consequence of deaths from e-cigarettes: this model allows for e-cigarettes to increase the population of individuals who use any of any tobacco product (combustible cigarettes or e-cigarettes) and consequently the total deaths from e-cigarettes. This is consistent with previous research on the trade-off between the prevalence of use and the risk profile of a product: that is, a greater number of individuals who use a product are allowable from a public health perspective when using a less-risky product [64].

These results should be continually reconsidered with relevant external events and policies that impact the system. For example, the USA recently increased the legal age to purchase combustible cigarettes from age 18 to 21, and this is not reflected in the current model. If the increase in purchasing age has its intended effect, the smoking prevalence will decrease beyond what is accounted for in the current model. Future improvements to the model can take into account such external changes, especially if forthcoming literature is able to quantify the effects on smoking initiation and other variables in the system.

### Limitations

This study should be interpreted in the context of important limitations. The central limitation of system dynamics modeling is that the results may not reflect what occurs in reality; some models can produce the right historical behavior for the wrong reasons (i.e., the wrong model structure), making future projections inaccurate. However, the series of validation tests performed increase confidence that this model captures the relevant causal relationships in the real-world system. Though additional elements can be added to the model, parsimony is desirable once the minimally essential features have been captured. A specific simplifying assumption was excluding individuals who formerly used e-cigarettes from the model. Since the stocks relevant to tobacco-related death and disease are in relation to individuals who use e-cigarettes, excluding a stock for individuals who formerly used e-cigarettes has the effect of assuming individuals who currently use e-cigarettes remain at the same risk for life. Thus, this is a conservative assumption that likely overestimates the mortality from established e-cigarette use.

Other model assumptions are based on imperfect data and impact the magnitude and trends of e-cigarette initiation rates and stocks of established numbers of smokers and individuals who use e-cigarettes. In particular, the strength of the diversion, smoking reduction, and cessation effects are based on estimates from the current literature, which may be limited. The diversion effect is particularly controversial, as much existing literature has argued for a gateway effect of e-cigarettes, which is an opposing effect. However, recent studies show that population-level trends are inconsistent with a gateway account, as the combustible cigarette smoking prevalence continues to decline and may even have accelerated after the introduction of e-cigarettes [39, 40]. This suggests a net diversion effect [43], the magnitude of which is estimated based on the diversion effect necessary to account for the accelerating decline in smoking after e-cigarettes appeared [65]. The current system dynamics model will be updated as new data emerge.

Additional limitations of the model include the focus on only two tobacco products (combustible cigarettes and e-cigarettes). Other products may alter the dynamics presented here, especially with cigar use surpassing combustible cigarette use among youth [66]. Similarly, the current policy was limited to overturning existing e-liquid flavor bans and delivering an informational campaign; however, additional policies could alter the findings, such as altering existing age restrictions on purchasing e-cigarettes, which is a different method of restricting access to e-cigarettes. Additional implementation challenges that were not included in the model may also be relevant and would have the effect of delaying and weakening the policy effects.

The model is also limited to the policy environment and e-cigarette market in the USA at the time of this research. Both are in flux and will continue to be in coming years. Specifically, the age of tobacco purchase has recently been raised to 21 years in the USA as of December 2019, and e-cigarette regulations have been implemented in several states and localities in the USA. The broad class of e-cigarette products has evolved across several generations of devices since their invention, and possible improvements to e-cigarette technology may warrant changes in model parameters (e.g., reducing potential e-cigarette deaths under this pessimistic scenario). Another limitation is that, while the model incorporates some elements of harm perceptions (i.e., the time delay in updating perceptions), harm perceptions are not explicitly modeled; however, harm perceptions likely have an important effect on use. Further, harm perceptions (and misperceptions) of e-cigarettes are sensitive to media messaging, for example the EVALI scare in the USA in 2019. Future research could explore the effects of recent policies enacted in the USA and could model other policies and implementation challenges in more detail. Additionally, future work could incorporate changes in the e-cigarette market, as well as consider the impact of “shocks” to (mis)perceptions of e-cigarettes’ relative risk compared to combustible cigarettes.

### Strengths

The current study is novel in its examination of a harm reduction policy promoting e-cigarettes, particularly with respect to 3 possible mechanisms by which e-cigarette use can decrease the combustible cigarette smoking prevalence. The question of diversion, or primary prevention of combustible cigarette use, is particularly novel, as this is a difficult effect to estimate empirically and has thus been understudied to date [41]. Additionally, the use of system dynamics modeling allows for a systematic examination of different scenarios, ranging from the status quo (no policy) to an ideal-world policy, as well as a range of realistic scenarios in between that present obstacles and delays to policy implementation. The current model has its focus on practical considerations with respect to policy implementation, ranging from time delays to political resistance to budgetary limitations. Finally, the model is publicly available via a user-friendly web interface [59] as well as a downloadable full model [52], allowing further testing of policy scenarios and assumptions of the model.

## Conclusions

The system dynamics simulation model presented predicts that promoting e-cigarettes as a less harmful alternative could help reduce the smoking prevalence and consequently the smoking attributable deaths. Findings show that even under a worst-case assumption in which e-cigarette use contributes to total mortality, the proposed harm reduction policy promoting e-cigarettes in lieu of combustible cigarettes is predicted to drastically reduce the tobacco-related disease burden. Specific projections will change based on different assumptions, parameters, and implementation logistics, and ongoing modeling work can incorporate additional hypothetical scenarios.

## Data Availability

The full simulation model is downloadable from: https://exchange.iseesystems.com/models/player/arielle-selya/e-cigarette-harm-reduction-policy. This simulation model can be viewed (including model structure, equations, and documentation) and run using the free software isee player [53]. A user-friendly, interactive web interface is available at: https://exchange.iseesystems.com/public/arielle-selya/e-cigarette-harm-reduction-policy-interface-tool

## Abbreviations

CLD: Causal loop diagram
FDA: Food and Drug Administration
NRT: Nicotine replacement therapy
NYTS: National Youth Tobacco Survey
